# Manitoba Veterinary Medical Association

**Published:** 1896-02

**Authors:** W. J. Hinman

**Affiliations:** Secretary and Registrar


### MANITOBA VETERINARY MEDICAL ASSOCIATION.

THE semi-annual meeting was held on December 17, 1895, Dr. Young, Vice-President, in the chair. Those present were Messrs. S. J. Thompson, W. Sumerton, D. J. McGillivray, C. A. Turley, C. A. Harrison, J. Lipsett, J. G. Rutherford, W. A. Dunbar, W. J. Hinman, R. Price, of St. Paul, Minn.

The minutes of the former meeting were adopted.

*Resolved*, upon motion, That all members in arrears be removed from the register and published lists. The furnishing of agricultural papers with veterinary matter was condemned by the members. Dr. Torrance, the President, was prevented from attending on account of the death of his brother, and a resolution of sympathy was ordered sent him.

*Resolved*, Rutherford, Hinman, That the Secretary be instructed to communicate with the Western Veterinary Association, and to proffer that body the hearty sympathy and co-operation of the Veterinary Association of Manitoba in its timely efforts to elevate the status of the profession by lengthening the curriculum, and raising the standard of the matriculation, and final examination of the Ontario Veterinary College.

*Resolved*, That in view of the rapid strides with which the veterinary profession has recently advanced, and the increased amount of scientific knowledge rendered necessary thereby, it is advisable that the Ontario Veterinary College should make an effort to keep abreast of the times; that therefore applicants for admission to the college be required to pass a matriculation examination, which shall demonstrate their ability to read and write the English language correctly, and to understand the rules of arithmetic as far as decimal fractions; that the matriculation examination be conducted by a board of examiners entirely apart from and independent of the faculty of the college; that the curriculum be extended to three sessions of six months each, and that students be required to practise at least four months of each vacation with a duly qualified practitioner; that the examination for the diploma shall be upon the practical as well as the scientific attainments of the candidates; that the examiners be elected by the graduates of the college resident in Canada, in the manner similar to that in which the members of the Council of the Royal College of Veterinary Surgeons are chosen; that the Secretary be instructed to forward copies of these resolutions to Professor Smith, to Mr. Henry Wade, Secretary of the Arts and Agricultural Association, and to the various veterinary periodicals. These motions were carried.

*Resolved*, That at the next session of the Legislature of Manitoba this Association should ask for an amendment to the Veterinary Association Act of 1890, whereby sub-section (c) of section 4 shall be made to apply only to graduates of schools having a curriculum of at least three sassions of six months each, and striking out the words "in any part of Her Majesty's Dominions," where they occur in the aforesaid sub-section.

Dr. Price explained the Minnesota Veterinary Act, and considered this Province as one superior to any country in the world.

The subject of "Tuberculosis and the Tuberlin Test" was discussed at great length. Dr. Price, of St. Paul, opened the discussion, and was followed by Dr. Rutherford, of Portage la Prairie. (These papers will be published in the March JOURNAL.)

Dr. Odinman, Dairy Inspector for the city of Winnipeg, explained the working of the Act and the by-laws relating to the tuberculin test and tuberculosis. He thoroughly and firmly believed in the efficacy of the test and its reliability. He had injected over 1200 head, and had found very few cases whereupon post-mortem the test had failed to denote the condition of the animal. He explained shortly the Act, saying it was compulsory on all persons selling milk in the city to have their cows tested. They were charged a license of 50 cents per cow, and he was paid 50 cents per head for applying the test, the government furnishing the tuberculin. Those reacting were branded on the left horn and those not on the right one with a number, and the milksellers instructed to at once isolate completely the diseased ones from his premises. He believed that in 90 per cent. the test properly administered was accurate.

Dr. Dunbar believed that two great importance was placed upon the contagious nature, and would not hesitate in drinking milk from tuberculous animals, but agreed with the other speakers as to the nature of the disease. He considered there was a tendency to cause too much excitement over the matter. He would like to see some reasonable and satisfactory arrangements made for the disposal of the animals condemned by the tuberculin test—a test in which he had confidence, and which he considered as good as any other test which had been discovered. He was in sympathy with the Association in its endeavors to bring about the eradication of the disease.

Dr. Young, having given an account of his personal experience with the disease, also as an inspector at the boundry, strongly recommended the test.

Dr. Thompson, Provincial Veterinarian, gave an excellent address upon the value of the test, the nature of the disease, and his experience. He strongly advocated the test, and hoped soon to see the government take the matter up as had the city of Winnipeg.

The counsel of the Association was instructed to prosecute all unqualified practitioners, which means all graduates in arrears to the Association, any persons practising as veterinary surgeons whose names are not on the register. Several other important matters were disposed of when the meeting adjourned.

W. J. HINMAN.
Secretary and Registrar.

